# Atrial flutter in a patient with atrial septal defect and *anomalous venous drainage*: unusual approach for ablation

**DOI:** 10.1002/ccr3.998

**Published:** 2017-05-23

**Authors:** Ivo Roca‐Luque, Nuria Rivas, Laura Dos, Jaume Francisco, Jordi Pérez‐Rodon, Antònia Pijuan, David Garcia‐Dorado, Àngel Moya

**Affiliations:** ^1^ Arrhythmia UnitCardiology DepartmentVall d'Hebron Universitary HospitalBarcelonaSpain; ^2^ Grown‐Up Congenital Heart Disease UnitCardiology DepartmentVall d'Hebron Universitary HospitalBarcelonaSpain; ^3^ Cardiology DepartmentVall d'Hebron Universitary HospitalBarcelonaSpain

**Keywords:** Ablation, atrial flutter, congenital heart disease, vascular abnormalities

## Abstract

Atrial flutter ablation in CHD (Congenital Heart Disease) patients is a challenging procedure because of the possibility of multiple circuits. Electroanatomical mapping and pacing maneuvers are crucial to determine critical isthmus. Moreover, vascular abnormalities and residual cardiac defects need to be known before the ablation to decide the better strategy for ablation.

## Introduction

Atrial flutter is common in patients with surgically corrected congenital heart diseases. In these patients, anatomical abnormalities caused not only by congenital cardiac defect but also by surgical correction make the diagnosis and ablation more challenging. Moreover, vascular abnormalities related to cardiac disease can make the procedure even more difficult.

## Case Report

This is a 70‐year‐old female patient that was referred to our grown‐up congenital heart disease outpatient clinic because of dyspnea grade II and palpitations. She referred that she had underwent surgical correction of a sinus venous atrial septal defect (ASD) with partial anomalous pulmonary venous connection at the age of 47 in another institution. She did not have any additional information about this operation.

**Figure 1:**
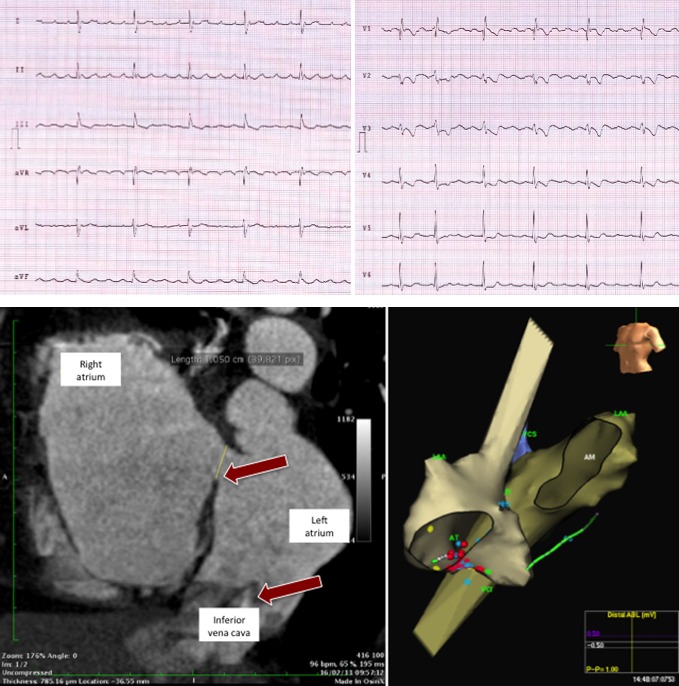
Upper panel shows surface ECG with atrial flutter, positive F waves in inferior leads. Bottom left panel: CT scan with residual ASD (upper arrow) and with anomalous drainage of inferior vena cava (bottom arrow). Bottom right panel: LAO view of electroanatomical reconstruction of both atria, confirming drainage of IVC into left atria.

Physical examination showed mild peripheral cyanosis on rest, without signs of congestive heart failure. Baseline ECG (Fig. 1.) showed an atypical atrial flutter with predominantly positive F waves in DII, aVF, V3‐V6, variable AV conduction, and a right bundle branch block (RBBB) pattern. Transthoracic echocardiogram suggested the presence of residual ASD. A CT scan was performed and confirmed (Fig. 1) both a residual ASD and an anomalous drainage of the inferior vena cava (IVC) to the left atrium. These findings explained the cyanosis and also suggested that the main lesion was not only an ASD, but also an incomplete total anomalous venous drainage and ASD associated and, for unknown reasons, correction of anomalous drainage of IVC had not been performed during surgery.

**Figure 2:**
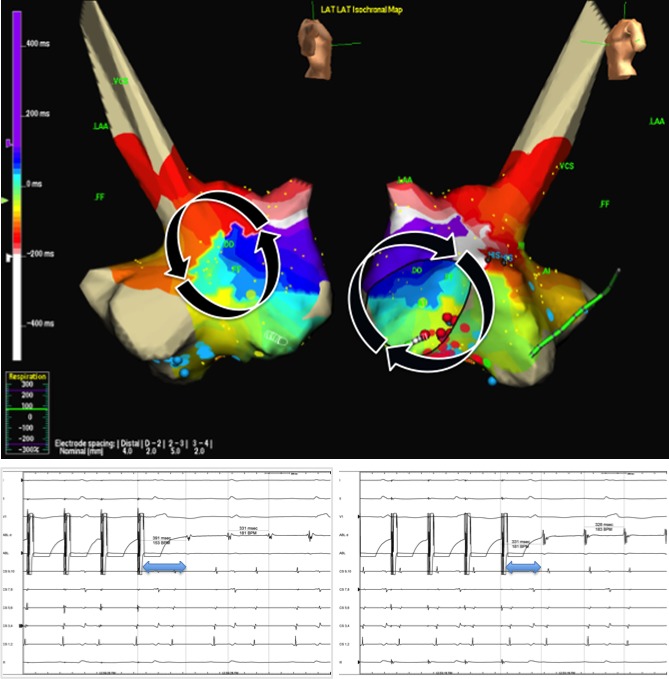
Upper panel shows activation map of both right and left atria. Activation of left atrium is passive and two different circuits are suggested: one counterclockwise circuit around lateral scar (left) and a clockwise circuit around tricuspid annulus (right). Left bottom panel shows entrainment from border zone of lateral scar with PPI‐TCL >30 msec (91 msec) excluding this area as a critical isthmus of clinical flutter. Right bottom panel shows entrainment from cavotricuspid isthmus with PPI‐TCL < 30 msec (3 msec) and concealed fusion, confirming that cavotricuspid isthmus is the protected isthmus of the circuit.

**Figure 3:**
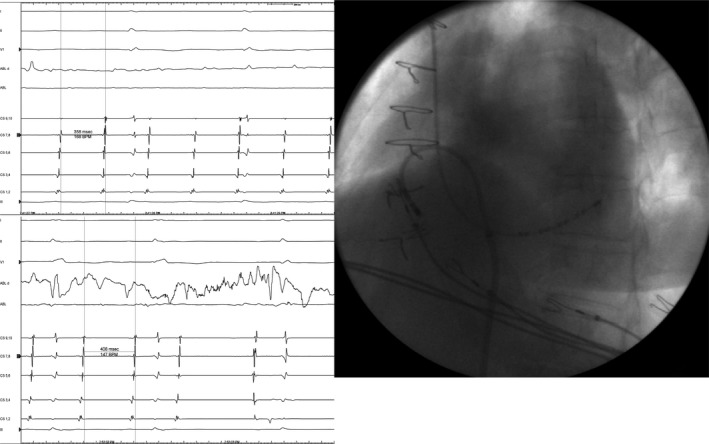
Upper left panel shows slowing of TCL from 331 to 358 msec during ablation in posterior part of cavotricuspid isthmus through. Bottom left panel shows slowing TCL from 358 to 409 msec and stop of flutter during ablation of a gap area of anterior isthmus, through IVC and residual ASD (catheter positioned as showed in fluoroscopy image).

The procedure was performed using NavX^®^ Ensite (Endocardial Solutions, St. Paul, MN) as an electroanatomic mapping system. A decapolar catheter (Inquiry 5F, St Jude Medical.) was advanced within the coronary sinus (CS) through the left subclavian vein. The right jugular vein was punctured for accessing the right atrium, the right femoral vein was punctured to access the left. An irrigated‐tip catheter (Biosense Webster Thermocool 4 mm, 7F) was used for mapping and ablation. A complete electroanatomical map from both atria (Fig. 1) was obtained. Activation map showed counterclockwise activation around the lateral scar of the right atria and clockwise activation around the tricuspid annulus with slow conduction in the anterior cavotricuspid isthmus (Fig. 2), with passive activation of left atria. Double and fractionated potentials were observed both in lateral right atria and in the septum, probably related to the atriotomy scar and the patch used to close the ASD. Entrainment maneuvers showed long postpacing interval (PPI) in the lateral wall and PPI identical to tachycardia cycle length (TCL) with concealed fusion in the cavotricuspid isthmus. These features confirmed that the critical circuit was the clockwise flutter around tricuspid annulus (Fig. 2). Because the anomalous IVC drainage into the left atrium, the cavotricuspid isthmus involved both atria so ablation in both sites was needed to achieve isthmus block. We started radiofrequency ablation through the jugular vein in the right atria, but no changes in TCL or activation pattern were observed. During ablation of the posterior part of the cavotricuspid isthmus in the left atria through the femoral vein, an increase of the TCL occurred without an interruption of the flutter. Finally, we advanced the ablation catheter inserted through right femoral vein from left atrium to right atrium through residual ASD and managed to reach the part of the cavotricuspid isthmus that was difficult to reach through the jugular access. During ablation in that area, the flutter was successfully interrupted (Fig. 3). We continued ablation until bidirectional block was achieved.

## Discussion

Atrial flutter is a common and potentially severe complication in patients that have undergone surgical correction of any congenital heart disease 1. Although scar‐related flutters are more frequent in these patients, most series show the isthmus‐dependent flutter as the most common macroreentrant arrhythmia 1, 2. Electroanatomical mapping is crucial in these patients, not only because it provides a map of the scar and depicts the channels and slow conduction areas that may be involved in the diastolic pathway of flutter 3, but also defines the ablation lines between scars and the anatomical and/or functional barriers. However, because scars or block lines can be multiple, as in our patients, activation maps sometimes show two different circuits. Therefore, pacing maneuvers are always needed to determine the critical pathway, which is often related to cavotricuspid isthmus, even if a scar‐related circuit is suggested after obtaining the activation map. In addition to the complex diagnosis of these flutters, anatomical difficulties are frequent in these patients, due to both cardiac and vascular anomalies. In this sense, surgically excluded cavotricuspid isthmus has been well described in patients who have previously undergone atrial switch procedures for great vessel transposition 4 and other complex congenital heart diseases. Ablation in both atria is often needed to achieve isthmus bidirectional block in these cases. However, few cases of anomalous or IVC interruption in patients submitted to ablation procedures have been reported 5, 6, 7 and most of them reach the right atrial through a superior access or through the azygos vein. In our case, probably because the patient had an anomalous IVC drainage unnoticed during ASD surgery, both atria had to be reached for ablation because the cavotriscupid isthmus had been surgically excluded. Despite the technical difficulties, the anomalous IVC drainage and residual ASD allowed us to access the right atria through the femoral vein. With this approach we managed to arrive in a crucial area of cavotricuspid isthmus that was difficult to achieve from superior access. In that area, flutter was interrupted and isthmus bidirectional block was achieved. In our opinion, complete ablation would have never been feasible from a superior access. In any case, complex anatomy in these patients should be carefully studied before the procedure to understand anatomical relationships, to plan the ablation approach carefully and, sometimes, to use the anomalies to facilitate ablation procedure. This case reflects the difficulties in diagnosis and ablation of atrial flutter in patients with surgically corrected congenital heart disease. However, because in this population atrial arrhythmias are related to worse prognosis, early invasive treatment is recommended despite ablation procedure might be challenging.

## Authorship

IR: conceived and designed the study. IR, NR: drafted the manuscript. IR, JF, JP, NR: acquired the data. LD, AP: revised the manuscript for key content in field of congenital heart disease. AMM, DGD: made critical revision of the manuscript for key intellectual content.

## Conflict of Interest

None declared.

## References

[1] Triedman, J. K. 2002 Arrhythmias in adult patients with congenital heart disease. Heart 87:383–389. 11907020 10.1136/heart.87.4.383 PMC1767082

[2] Chan, D. , G. Van Hare , J. Mackall , M. Carlson , and A. Waldo . 2000 Importance of atrial flutter ishtmus in postoperative intra‐atrial reentrant tachycardia. Circulation 102:1283–1289. 10982544 10.1161/01.cir.102.11.1283

[3] Triedman, J. K. , M. Alexander , C. Berul , L. Bevilacqua , and E. Walsh . 2001 Electroanatomical mapping of entrained and exit zones in patients with repaired congenital heart disease and intra‐atrial reentrant tachycardia. Circulation 103:2060–2065. 11319195 10.1161/01.cir.103.16.2060

[4] El Yamman, M. , S. Asirvatham , S. Kapa , R. Barrett , D. Packer , and C. B. Porter . 2009 Methods to access the surgically excluded cavotricuspid isthmus for complete ablation of typical atrial flutter in patients with congenital heart defects. Heart Rhythm 6:949–956. 19482557 10.1016/j.hrthm.2009.03.017

[5] Guenther, K. , M. Marrouche , and J. Ruef . 2007 Ablation of atrial flutter by the femoral approach in the absence of inferior vena cava. Europace 2007; 9: 1073–1074. 17673496 10.1093/europace/eum150

[6] Pai, R. K. , J. F. MacGregor , M. Abedin , and M. H. Hamdan . 2005 Case report: radiofrequency catheter ablation of typical atrial flutter and the atrioventricular junction via the superior vena cava approach in a patient with a congenital absence of an inferior vena cava. J. Interv. Card. Electrophysiol. 14:193–195. 16421696 10.1007/s10840-006-5456-2

[7] Pérez‐Silva, A. , J. L. Merino , R. Peinado , and J. Lopez‐Sendon . 2011 Atrial flutter ablation through the azygous continuation in a patient with inferior vena cava interruption. Europace 13:442–443. 21030393 10.1093/europace/euq385

